# Capillary‐Based Physicochemical Characterization of Lipid Nanoparticles

**DOI:** 10.1002/elps.70032

**Published:** 2025-09-28

**Authors:** Evrim Ümit Kuzucu, Valentin Schittny, Jörg Huwyler, Maria Anna Schwarz

**Affiliations:** ^1^ Division of Pharmaceutical Technology Department of Pharmaceutical Sciences University of Basel Basel Switzerland; ^2^ Solvias Kaiseraugst Switzerland

**Keywords:** capillary zone electrophoresis (CZE) | critical quality attributes | electrohydrodynamic coupling | electrophoretic Taylor dispersion (eTD) | gene therapy | lipid nanoparticles | nucleic acids | Taylor dispersion (TD)

## Abstract

Lipid nanoparticles (LNPs) are widely used for the delivery of nucleic acid (NAs), most notably in gene therapy and messenger ribonucleic acid (mRNA)‐based vaccines. Understanding their physicochemical properties is essential, yet current analytical approaches often fall short in capturing their complexity. Here, we introduce an analytical strategy using capillary zone electrophoresis (CZE) and pressure‐driven Taylor dispersion (TD) analysis beside the combination of both separation principles. This novel separation mode of electrophoretic TD or electrohydrodynamic coupling (termed here as eTD) can be used to characterize deoxyribonucleic acid (DNA)‐loaded LNP formulations using standard capillary electrophoresis (CE) instrumentation. eTD is a new separation approach that combines electrophoretic and hydrodynamic movement in micro‐scaled capillaries for the analysis of drug carriers as LNPs. Focusing on critical quality attributes (CQAs), TD provided information on the hydrodynamic radius of LNPs and the distribution of NAs across different chemical environments. CZE enabled the estimation of ζ‐potential and localization of DNA within distinct particle populations. The novel eTD mode offers deeper insight into LNP structure and morphological aspects, yielding characteristic profiles for individual formulations and revealing the presence of unencapsulated DNA. To contextualize LNP measurements, we also analysed free NAs and their mixtures with LNPs under identical conditions. The method distinguished between encapsulated and unencapsulated species, revealing individual electrophoretic and dispersion profiles for single‐stranded mRNA and double‐stranded DNA. These findings demonstrate the potential of capillary techniques for the advanced physicochemical characterization of NA‐loaded LNPs. Further investigations are warranted to expand their analytical utility and deepen our understanding of LNP structural features.

**Abbreviations**
BGE: background electrolyte
CE: capillary electrophoresis
CIEF: capillary isoelectric focusing
CQA: critical quality attribute
Cryo‐TEM: cryogenic transmission electron microscopy
CZE: capillary zone electrophoresis
DLS: dynamic light scattering
ds: double‐stranded
ELS: electrophoretic light scattering
EOF: electroosmotic flow
eTD: electrophoretic Taylor dispersion or electrohydrodynamic coupling
GCE: capillary gel electrophoresis
HPLC: high performance liquid chromatography
IP: ion‐pairing
LIFl: aser‐induced fluorescence
LNP: lipid nanoparticle
mRNA: messenger ribonucleic acid
NA: nucleic acid
np: normal polarity
NR: NileRed
p: pressure
PDI: polydispersity index
pDNA: plasmid DNA
RFU: relative fluorescence units
RNA: ribonucleic acid
rp: reversed polarity
ss: single‐stranded
TD: Taylor dispersion

## 1 Introduction

Lipid nanoparticles (LNPs) are used for the delivery of deoxyribonucleic acid (DNA) and ribonucleic acid (RNA) in gene therapy and for vaccines. Their potential has been demonstrated by the FDA approval of the messenger RNA (mRNA)‐based SARS‐CoV‐2 vaccine [1]. The effectiveness of LNP‐based therapeutics is linked to the precise characterization of their physical and chemical properties, which determine key performance attributes such as stability, cellular uptake and transfection efficiency. To achieve optimal formulation, various physicochemical analytical techniques are used to evaluate defined critical quality attributes (CQAs) [2].

**FIGURE 1:**
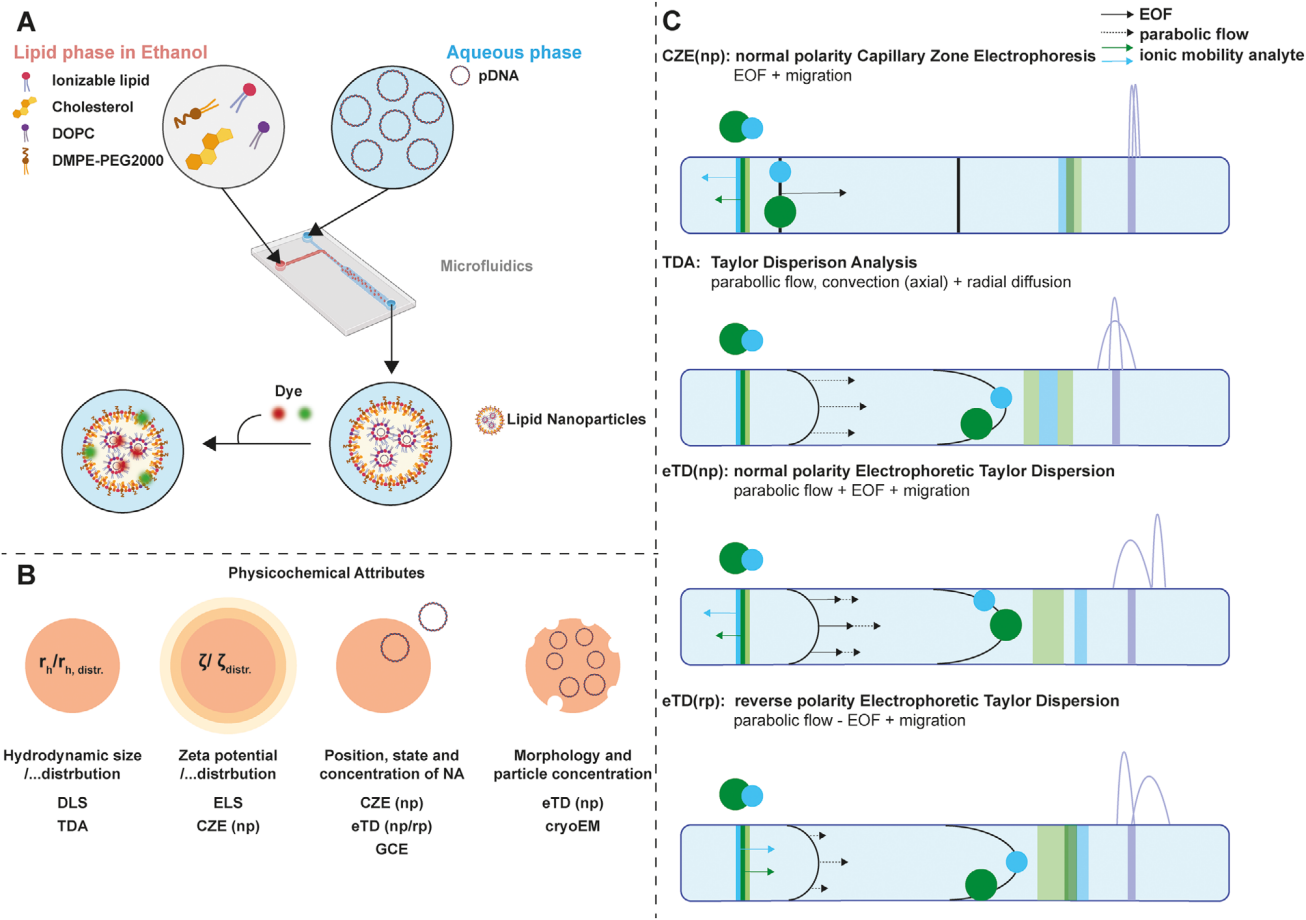
Manufacturing process, physicochemical attributes and corresponding state‐of‐the art and novel analytical tools. (A) Schematic representation of the production process for the studied LNPs, subsequently labelled with DNA‐ or lipid‐specific dyes. (B) Overview of physicochemical attributes along with potential state‐of‐the art or novel analytical tools for their characterization. (C) Separation principles considering CZE(np), TD and eTD. eTD can be operated under normal (np) or reversed (rp) polarity. Arrows indicate the particles’ intrinsic ionic mobility and the flows generated by pressure or electric field. Green and blue spheres represent particles differing in their charge and size. CZE, capillary zone electrophoresis; DLS, dynamic light scattering; DOPC, 1,2‐dioleoyl‐sn‐glycero‐3‐phosphocholine; ELS, electrophoretic light scattering; EOF, electroosmotic flow; eTD, electrophoretic Taylor dispersion or electrohydrodynamic coupling; GCE, capillary gel electrophoresis; NA, nucleic acid; pDNA, plasmid DNA.

LNP–nucleic acid (NA) formulations present significant challenges due to their complexity and thus the number and definition of CQA (Figure 1B), distinguishing them from other biologics such as protein therapeutics. Key physicochemical CQAs include particle size and size distribution, which are closely linked to particle composition and its intrinsic zeta potential (ζ‐potential), defining the ζ‐distribution. In addition, the structural state and localization of the cargo are important parameters. Assessing cargo integrity is particularly challenging and includes NA fragmentation, loading efficiency per particle and the folding conformation. Furthermore, micellar or micelle‐like structures formed independently of the primary LNPs may also associate with cargo molecules, leading to reversibly associated but unprotected NA assemblies. Currently, only a limited number of analytical tools are available to detect and characterize such heterogeneous features. Although this issue has been acknowledged in literature, a robust and comprehensive analytical solution remains elusive [3].

Dynamic light scattering (DLS) is among the most used methods for determining the hydrodynamic diameter and polydispersity index (PDI) of LNPs [4]. However, several inherent limitations must be considered: (i) Size estimations are disproportionately influenced by larger particles, potentially skewing the results in polydisperse samples; and (ii) although the hydrodynamic radius derived from the Stokes–Einstein equation is based on an equivalent sphere model, it may not accurately represent the true size or morphology of non‐spherical or structurally heterogeneous nanoparticles [5]. The PDI is a critical parameter for assessing the uniformity of LNP formulations, with lower PDI values indicating more homogenous particle populations. In addition to size, electrophoretic light scattering (ELS) is commonly used to measure the ζ‐potential, which reflects the electrokinetic potential at the shear plane of nanoparticles in suspension.

Cryogenic transmission electron microscopy (cryo‐TEM) is a widely used technique for analysing the morphology of LNPs. This technique allows direct visualization of LNPs in a near‐native, vitrified state, providing high‐resolution images, revealing intricate structural details without artefacts introduced by chemical fixation or staining [6]. Cryo‐TEM is invaluable for verifying particle size, shape and structure, all of which are critical for optimizing LNP formulations and ensuring the stability and functionality of therapeutic systems.

To evaluate DNA encapsulation efficiency, the NA exclusion assay using SybrGold is a commonly applied fluorescence‐based method [7]. It relies on the selective binding of SybrGold to DNA, allowing the quantification of accessible NAs before and after disruption of the LNP. However, several limitations compromise its reliability: (i) The dye may partially permeate intact LNPs, leading to overestimated fluorescence signals and misrepresentation of unencapsulated DNA; (ii) the use of denaturing agents such as Triton X‐100 generates a chemically undefined mixture of lipids, NAs and surfactants, potentially affecting dye specificity and sensitivity [8]. Therefore, in the present study, Triton X‐100 was substituted by the organic solvent propanol.

The quantification of NAs by ion‐pairing‐RP (IP‐RP), (IP) anion exchange chromatography ((IP)‐AEX), hydrophilic interaction chromatography (HILIC) or capillary gel electrophoresis (CGE) [9] typically requires the complete break‐up of the LNP formulation, as does the quantifications/characterization of the lipids by high‐performance liquid chromatography (HPLC) using a charge aerosol detector (CAD) [10]. As a result, these approaches do not preserve the system's chemical equilibrium, limiting insights into the formulation's native state. In contrast, capillary‐based methods operating without gel matrix background electrolyte (BGE) enable the analysis of intact and fully assembled LNPs by monitoring their migration, dispersion or elution behaviour within well‐defined microchannels, while maintaining their structural and physicochemical integrity.

Devices designed for capillary electrophoresis (CE) offer favourable conditions for the analysis of nanoparticles and macromolecules. This is due to the use of micrometre‐scale capillaries, operation under chemical equilibrium, the ability to combine electroosmotic and pressure‐driven flows and the integration of online detection systems. From this it can be deduced that classic CE devices are generally suitable for the three modes described here, namely, electrophoretic modes such as capillary zone electrophoresis (CZE), pressure‐driven modes Taylor dispersion (TD) and the combination of both electrophoretic TD or electrohydrodynamic coupling (eTD). The latter is designated in the present publication as eTD.

It should be mentioned that the use of capillary‐based systems for LNP analysis remains poorly explored. This also applies to innovative solutions for the analysis of NAs. The few noteworthy works are outlined below. H. Ranchon et al. used hydrodynamic flow in viscoelastic fluids to induce transverse forces and thus separation of different DNA size variants [11]. Malburet et al. demonstrated the potential of combining TD and electrophoresis in a conventional CE setup to study RNA‐loaded LNP vaccines, using pressure‐driven mobilization and direct UV detection at 200 nm to assess particle size and surface charge [12]. In the experiments described there, a separation of an analyte containing LNPs at 0.1–0.4 psi (7–30 mbar) and 14 kV is shown, whereby LNPs were separated from a neutral marker as a single signal.

More recently, a CZE application using laser‐induced fluorescence (LIF) detection has been proposed as a promising method for the characterization of LNP‐based therapeutics [13]. Alternatively, a TD‐based application without the presence of an electrical field is described by Malburet et al. [14]. Different LNP formulations were compared using TD (UV) and DLS, but also free RNA was investigated, interestingly also treated with RNase. Thomas Le Saux and Herve’ Cottet present an innovative analytical approach for performing CZE separation followed by a subsequent TD mobilization with sample analytes such as DNA and copolymer [15]. It can be assumed that this type of combination of both separation principles is as informative as the electrohydrodynamic coupling shown here and should be considered for future developments in LNP analysis.

It was the aim of the present study to capture different separation modes using micro‐scaled capillaries combined with a specific online detection of NA‐containing analytes using standard CE instruments. This method enables the simultaneous assessment of LNP physicochemical properties, such as particle size, ζ‐potential and the spatial distribution of NAs under near‐equilibrium conditions. This will support the assessment of CQAs (Figure 1B). Rather than presenting a comprehensive analytical solution, this work aims to advance future developments in LNP formulation and characterization.

## 2 Materials and Methods

### 2.1 Materials

Lipid component (6*Z*,9*Z*,28*Z*,31*Z*)‐heptatriaconta‐6,9,28,31‐tetraen‐19‐yl‐4(dimethylamino)butanoate (MC3) was ordered from BroadPharm (Sandiego, CA). (4‐Hydroxybutyl)‐azanediyl‐bis‐(hexane‐6,1‐diyl)‐bis‐(2‐hexyldecanoate) (ALC‐0315) and heptadecan‐9‐yl‐8‐((2‐hydroxyethyl) (6oxo‐6‐(undecyloxy)hexyl)amino) octanoate (SM‐102) were obtained from MedchemExpress (Monmouth Junction, NJ). 1,2‐Dioleoyl‐sn‐glycero‐3‐phosphocholine (DOPC) and 1,2‐dimyristoyl‐sn‐glycero‐3‐phosphoethanolamine‐*N*‐[methoxy(polyethylene glycol)‐2000 (DMPE‐PEG2000)] were ordered from Avanti Polar Lipids (Alabaster, AL). Nanoplasmid DNA n.CAG.eGFP (3151 bp) was obtained by Nature Technology Corporation (Lincoln, NE). GFP mRNA was purchased from OZBiosiences (Cat. No. MRNA15C‐100, 918 nt). NaOH was obtained from VWR Chemicals (Radnor, PA). TBE buffer 10*×*, 10*×* Tris borate EDTA (TBE) buffer (890 mM Tris, 890 mM boric acid and 20 mM EDTA) was from Sigma‐Aldrich (St. Louis, MO). Nuclease‐free water, NileRed (NR), SybrGreen and SybrGold were purchased from Thermo Fisher Scientific (Waltham, MA). d(+)‐Glucose and 4‐(2‐hydroxyethyl)‐1‐piperazinethane‐sulphonic acid (HEPES) were ordered from PanReact AppliChem (Darmstadt, Germany).

### 2.2 LNP Production

LNPs were produced using the Automated Nanoparticle system (Particle Works, Royston, UK) via a microfluidic mixing approach. The organic phase consisted of ionizable lipids, cholesterol, DOPC and DMPE‐PEG‐2000 in a molar ratio of 50:38.5:10:1.5. These lipids were dissolved in sterile filtered 100% ethanol at a total lipid concentration of 2.95 mM. The aqueous phase, comprising 20 mM sodium acetate buffer (pH 4, sterile filtered), contained the NA constructs at a concentration of 20 µg/mL, yielding an N/P ratio of 6.

The two phases were mixed using a junction microfluidic chip with a depth of 190 µm at an organic‐to‐aqueous flow rate ratio of 1:3, with a total flow rate of 8 mL/min. The resulting LNP suspension was subjected to overnight (∼16 h) dialysis at 4°C using dialysis tubes (Millipore, 12–14 kDa molecular weight cut‐off) in dialysis buffer (0.9% NaCl, 1 mM HEPES, pH 7.4), under gentle stirring. After dialysis, the LNP samples were analysed using specified analytical techniques. In cases where a fluorescent labelling of the DNA was performed pre‐ or post‐production, a 1:10 000 SybrGreen concentration was used.

### 2.3 Analytical Methods

Physicochemical characterizations of LNPs were characterized as described previously [16]. The *r*_h_, PDI and ζ‐potential were determined by DLS using a Zetasizer Ultra instrument with the ZS Xplorer software (Malvern Panalytical, Worcestershire, UK). For ζ‐potential measurements, the LNPs were diluted in a 5 % glucose and 10 mM HEPES solution (sterile filtered). Cryo‐TEM was performed to study particle morphology.

### 2.4 CZE, TD and Electrohydrodynamic Coupling Analysis

Analysis was carried out using a SCIEX PA800 Plus system (Framingham, MA) equipped with a solid‐state laser with an excitation wavelength of 488 and a 520 or 560 nm band pass emission filter from Edmund Optics, a 30 kV power supply and a temperature‐controlled auto sampler (±2°C). In order to prevent electroosmotic flow (EOF), Neutral Capillary Conditioning Solution from SCIEX was used. Data were acquired using 32 Karat software 10.3 from SCIEX.

The BGE was 25 mM Tris, at pH 8.0, and 1:10 000 diluted SybrGreen. Analysis was performed in a 30/40 cm, 50 µm i.d. fused silica capillary at 10–30 kV with normal polarity (np) and rp. Prior to analysis, capillaries were conditioned for 1 min with 0.1 M NaOH (for np mode) and with conditioning solution (SCIEX) for 1 min (for rp mode). Sample injection was performed hydrodynamically at 1 psi for 10 s. If not specified otherwise, CE experiments were performed with the following standard settings: CZE(np): 30 kV, TD: 1.0 psi (69 mbar), eTD(np): 10 kV and 0.6 psi (41 mbar) and eTD(rp): −10 kV and 0.6 psi (41 mbar).

The LNP test sample solution was either directly injected or incubated with propanol (10%–50% v/v) for 10 min at room temperature. SybrGreen (1:100) of 10 % v/v was then added to both test sample solutions (standard approach). Using 10% v/v NR solution, the test sample solution was incubated for 10 min at 40°C. The handlings of the DNA or RNA test solution, as well the spiked LNP samples, were handled in the same way.

### 2.5 Plot Generation and Normalization

Data collection was performed using the SCIEX PA800 Plus system (500 Old Connecticut Path, Framingham, MA) at a sampling rate of 4 Hz. The recorded data were exported as ASCII (.asc) files and subsequently analysed using custom R scripts.

The R environment used for data processing and representation was R version 4.4.2. Key packages included the tidyverse suite (version 2.0.0), ggplot2 (version 3.5.1), lubridate (version 1.9.3), dplyr (1.1.4) and ggpubr (version 0.6.0), among others. Figures were designed with the help of the software packages BioRender and Adobe Illustrator.

CE‐traces were normalized with respect to relative fluorescence units (RFU) using a scale from 0 to 1 RFU. 0 RFU thereby corresponds to baseline signal and 1 RFU to the maximum observed absorbance. Maximum observed absorbance refers to either one individual CE‐trace (individual representation) or groups of several CE‐traces (group comparison).

### 2.6 Effective Mobility and ζ‐Potential Calculations

To determine the effective electrophoretic mobility and ζ‐potential of LNPs during CZE experiments, the apparent mobility μ was first calculated using the following equation:

$$\mu \left(\mathrm{LNP}\;\mathrm{or}\;\mathrm{EOF}\right)=\frac{v}{E}=\frac{l_{\mathrm{eff}}\cdot l_{\mathrm{tot}}}{t_{m}\left(\mathrm{LNP}\;\mathrm{or}\;\mathrm{EOF}\right)\cdot V}\left[\frac{m^{2}}{V\cdot s}\right]$$

where v represents the velocity of the analyte in m/s, and E stands for the electric field strength in V/m. $l_{\mathrm{eff}}$ and $l_{\mathrm{tot}}$ represent the effective and total capillary lengths of 0.3 m and 0.4 m respectively, whereas $t_{m}$ stands for the analyte migration time in s. V represents the applied voltage in V.

The effective mobility $\mu_{e}$ of the LNP was subsequently determined by subtracting the mobility of the EOF from that of the analyte, as shown in the following equation:

$$\mu_{e}=\mu_{\mathrm{LNP}}-\mu_{\mathrm{EOF}}\left[\frac{m^{2}}{V\cdot s}\right]$$

Finally, the ζ‐potential of a given LNP was calculated using the following equation:

$$\zeta =\frac{\mu_{e}\cdot \eta }{\varepsilon_{r}\cdot \varepsilon_{0}}\left[V\right]$$

where η=0.89mPa·s is the dynamic viscosity of water at 25°C [17], $\varepsilon_{r}=78.5$ represents the relative permittivity of water at 25°C [18] and $\varepsilon_{0}=8.85\cdot 10^{-12}\;\left[\frac{F}{m}\right]$ of a vacuum [19].

## 3 Results and Discussion

### 3.1 Working Hypothesis

The rationale and working hypothesis behind our experimental approach originated from the current need to characterize nanoparticles, specifically LNPs, and to be able to record their electrophoretic, electrodynamic and hydrodynamic properties. In addition to observing the movement of nanoparticles in an electric field, pressure‐based flow or a combination thereof (Figure 1C), visualization and detection are crucial for the analytical result. Using UV detection at 200 nm, all components of the analyte are detected nonspecifically, whereas the specific staining of the NA, presented here and subsequent LIF detection enables the targeted detection of all NA‐containing variants, an essential feature of the proposed analytical approach explained in this study.

### 3.2 Theoretical Considerations

The application of an electric field in CZE causes charged analytes to migrate at different speeds depending on their ratio of charge‐to‐hydrodynamic radius (*q*/*r*_h_) or, in the case of particles, the ratio of ζ/*r*_h_. The total velocity of the analyte is the sum of the bulk flow velocity, the EOF and the electrophoretic velocity of the analyte. Analytes, which have different *r*_h_ but the same *q*(ζ)/*r*_h_ ratio, cannot be separated by electrophoresis, for example, various RNA chains of different lengths. The typical characteristic of CZE is the flat flow profile, given by the EOF, and the slight broadening of the sample zone section according to the diffusion coefficient (*D*) of the individual analytes, beside other effects as adsorption, electrodispersion or overlapping of flow profiles. Here, we discuss the np mode to evaluate the CZE profile of free DNA/RNA and the corresponding migration behaviour of LNPs which are negatively charged at a pH of 8.

TD describes how an analyte spreads in a fluid flowing through a narrow capillary due to the combined effects of convection (bulk flow) and diffusion (molecular spreading) [5]. In laminar flow, a parabolic or Poiseuille‐like flow profile is generated, and thus, the velocity varies across the capillary diameter, being maximum at the centre and near‐zero at the walls. This velocity profile causes axial spreading of solutes, whereas diffusion smooths concentration gradients, leading to a characteristic Gaussian peak. The observed dispersion coefficient is related to molecular diffusion. Molecules with high diffusion coefficients exhibit less broadening of the sample zone to those with very low diffusion coefficients. However, both are detected at the same time with respect to the signal maximum. Relevant to notice, the initial width of the sample zone and the width of the detection window are significantly narrower than the sample zone achieved after dispersion. The longer the elution path, the broader the signals. In the context of discussion, it is worth noting that when capillary dimensions decrease into the lower micrometre range, molecules such as DNA or differently sized particles can be separated by the parabolic flow profile. This process, known as hydrodynamic chromatography (HDC), can occur in either a packed column or an open capillary. The effects observed in this study may, to some extent, overlap with those described for HDC investigations [20]. At operating parameters used here, 50 µm i.d. at a pressure of 0.6–1 psi (40–70 mbar) and particle sizes of up to 80 nm with a certain size heterogeneity, the separation conditions cannot be clearly assigned to the TD or HDC regime but rather to a transitional range [21]. Thus, we do not expect typical taylorgrams under the selected conditions. The interaction with an additional electric field (40 mbar and ±10 kV) and induced electrophoretic migration as well as EOF is described in the following section.

During electrohydrodynamic separation (eTD), both electrophoresis and TD occur simultaneously. Charged species migrate at different velocities based on their electrophoretic mobility, whereas TD broadens the solute zone according to molecular diffusion. Due to the lower pressure applied in eTD as compared to TD (1 psi) and the presence of the EOF, the peak broadening is significantly lower than in TD. Thus, the observed dispersion behaviour is influenced by the electrophoretic mobility *µ* (*q* (ζ)/*r*), but also from the individual *q* (ζ‐potential), *D* or *r*_h_, capillary dimensions as well as flow rate. The ionic migration of the DNA or lipid particle is in the opposite direction of the bulk flow in the np mode (Figure 1C, eTD(np)). In reversed polarity mode (eTD(rp)), the EOF is suppressed by a dynamic neutral coating, so analyte migration occurs solely by their own ionic mobility.

Furthermore, it seems that the ratio between applied voltage and pressure, the direction of the electric field and Poiseuille flow and the presence of an EOF play a key role for the phenomenon of transverse particle motion [22]. The results discussed in this study explain the interdependency of the applied electric field and the pressure gradient and subsequent particle cross‐section equilibrium position, sidewall or central focusing, of the particles within the microchannel and their size dependence, which were previously considered unknown. It is shown that for a specific condition the particle migration patterns can be altered by tuning the separation conditions, for example, the central focusing of the larger particles. Here, we present how this technique could be used for characterization of LNP systems, including both normal and rp.

### 3.3 Analysis of DNA and DNA‐LNPs by Different Capillary‐Based Protocols

The experiments presented in this study rely on the specific staining of NA and the subsequent observation of their movement within a microchannel (Figure 1A,C). The detection of various species in the microchannel is governed by their electrophoretic mobility and/or hydrodynamic motion, or a combination of both, and is monitored using LIF. Regarding staining, it is hypothesized that the fluorophore interacts with both the external DNA and DNA encapsulated due to permeation through the lipid bilayer accessing the internal particle sphere. Supporting this, a recent study by Brader et al. demonstrated that the cationic dye thionine can permeate into the LNP without disrupting its structural integrity [23]. Additionally, the fluorophore SybrGreen, utilized in this study, is known for its cell‐permeable properties, suggesting that it rapidly passes through the surface lipid bilayer [13].

The experiments utilized non‐covalent post‐staining with SybrGreen, a fluorophore that exhibits fluorescence only upon binding to NAs. With this staining approach, a separation profile of fluorescence signal pattern of all NA‐containing variants is generated that are accessible for the dye, including the NA located within the LNP. Theoretically, this allows the visualization of (i) NAs encapsulated within the LNP, (ii) those outside, either complexed or distributed in a micellar phase and (iii) in their free, unbound form.

It should be noted, different morphological structures of LNPs can occur within the formulation, from simple LNPs to multi blebbed LNPs, whereby the accessibility of the permeable fluorophore should then also change. These blebs are protrusions with an aqueous core surrounded by a lipid bilayer, separated from the solid lipid core [24]. Brader et al. showed that mRNA may be localized in blebs, if present, but in the case of spherical‐solid LNPs, mRNA is present below the surface lipid bilayer [23]. Even if both structures require the permeation of the dye through the lipid bilayer, different behaviours are to be expected.

To investigate whether the analysis of intact LNPs stained with the DNA‐specific dye SybrGreen effectively visualizes DNA‐containing lipophilic particles alongside other species, a comparative approach was employed using lipid‐specific dyes. Lipophilic dyes, such as NR, label the hydrophobic structures within the test sample preparation, providing a complementary perspective. This approach provides a consistent characterization of LNPs and phases, regardless of whether lipid‐specific dyes are used or DNA is visualized during LNP formulation or labelled according to standard procedure (Figure S1).

To analyse the total released DNA, the LNP‐containing test solutions were treated with propanol. Triton X‐100 is widely reported in the literature as a non‐ionic surfactant used at room temperature and/or elevated temperatures to disrupt LNPs [25]. Above its critical micelle concentration, Triton X‐100 forms micelles capable of dissolving the lipids derived from the LNP structure. However, it remains unclear in what form the DNA is subsequently present or whether it forms complexes with the mixed micellar structures. To address these uncertainties, our experiments incorporated propanol alongside Triton X‐100 to exclude potential side effects, such as interference with the interaction of SybrGreen with DNA.

**FIGURE 2:**
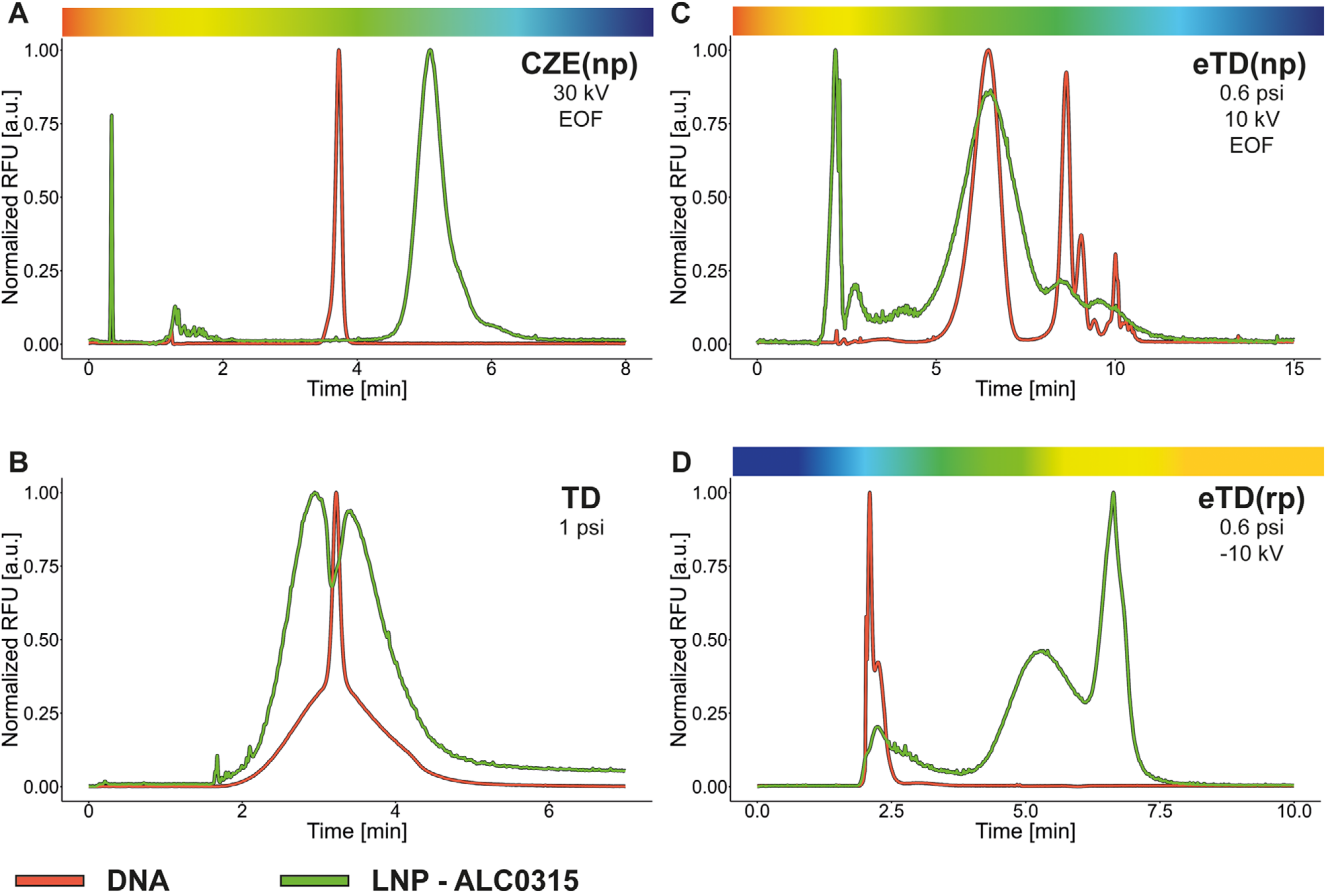
Analysis of DNA and DNA‐LNPs (ALC0315) by different microfluid‐based protocols. (A) CZE(np), (B) TD, (C) eTD operating under normal (np) or (D) reversed (rp) polarity were applied to analyse free DNA (red, 0.5–1 µg/mL) and DNA‐LNPs (green). Rainbow gradients indicate the approximate electric charge of the sample (red = cationic, blue = anionic and yellow = neutral). CZE, capillary zone electrophoresis; DNA, deoxyribonucleic acid; EOF, electroosmotic flow; eTD, electrophoretic Taylor dispersion or electrohydrodynamic coupling; LNP, lipid nanoparticle; RFU, relative fluorescence units; TD, Taylor dispersion.

**FIGURE 3:**
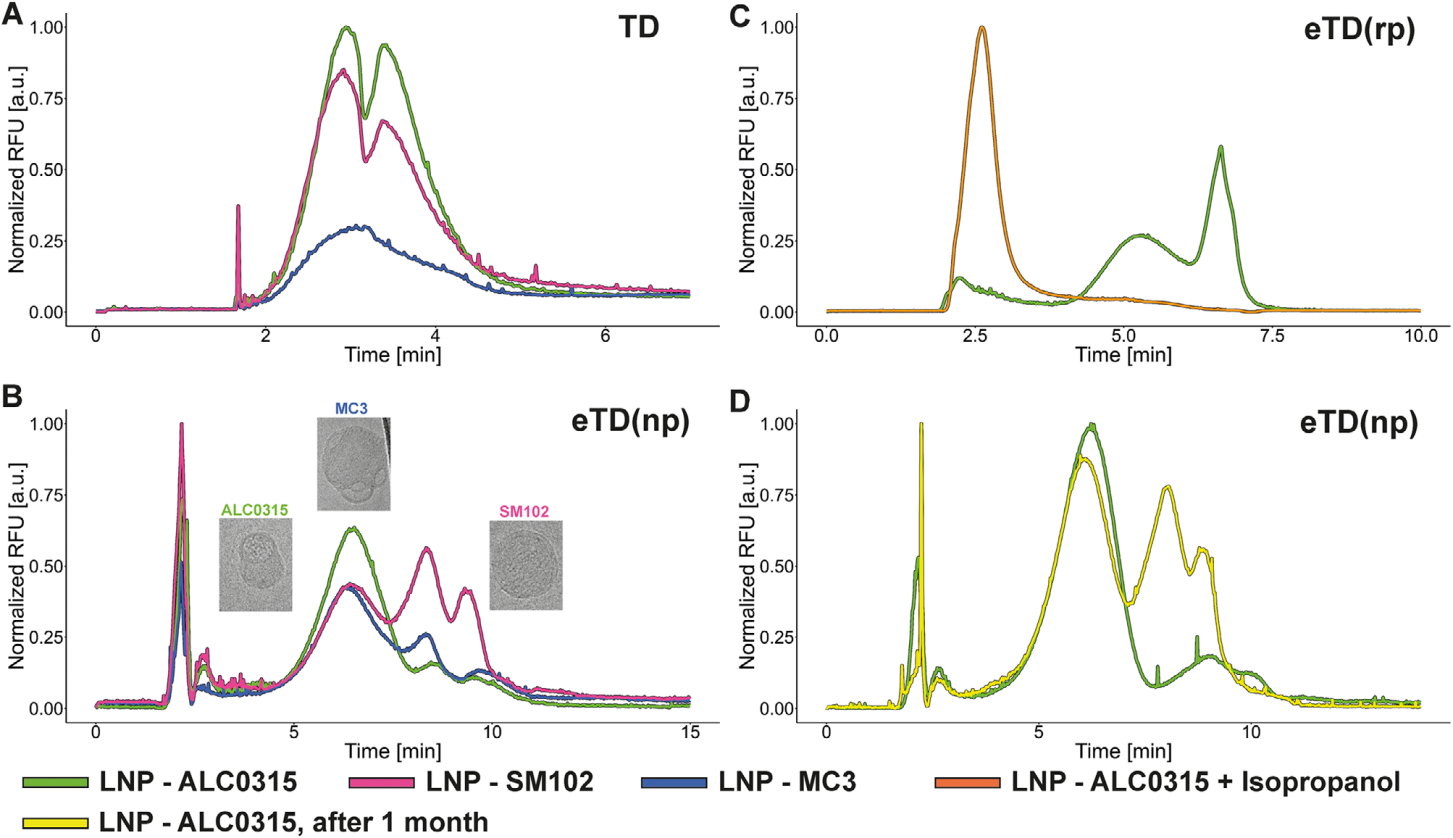
Evaluation of various DNA‐LNP formulations by TD and eTD, operated under normal (np) or reversed (rp) polarity. DNA‐LNPs formulated with ALC‐0315 (light green), SM102 (pink) or MC3 (blue) were analysed: (A) TD and (B) eTD analysis. Inserts display cryo‐TEM images of respective DNA‐LNP formulations. The horizontal dimension of the shown pictures corresponds to 100 nm. (C) Comparison of intact (dark green) and degraded (orange) DNA‐LNPs. (D) Comparison of freshly prepared (green) and aged (yellow; stored for 4 weeks at 4°C) DNA‐LNPs analysed by eTD(np). Please refer to Figure S9 for CZE(np) traces. eTD, electrophoretic Taylor dispersion or electrohydrodynamic coupling; LNP, lipid nanoparticle; RFU, relative fluorescence units; TD, Taylor dispersion.

**FIGURE 4:**
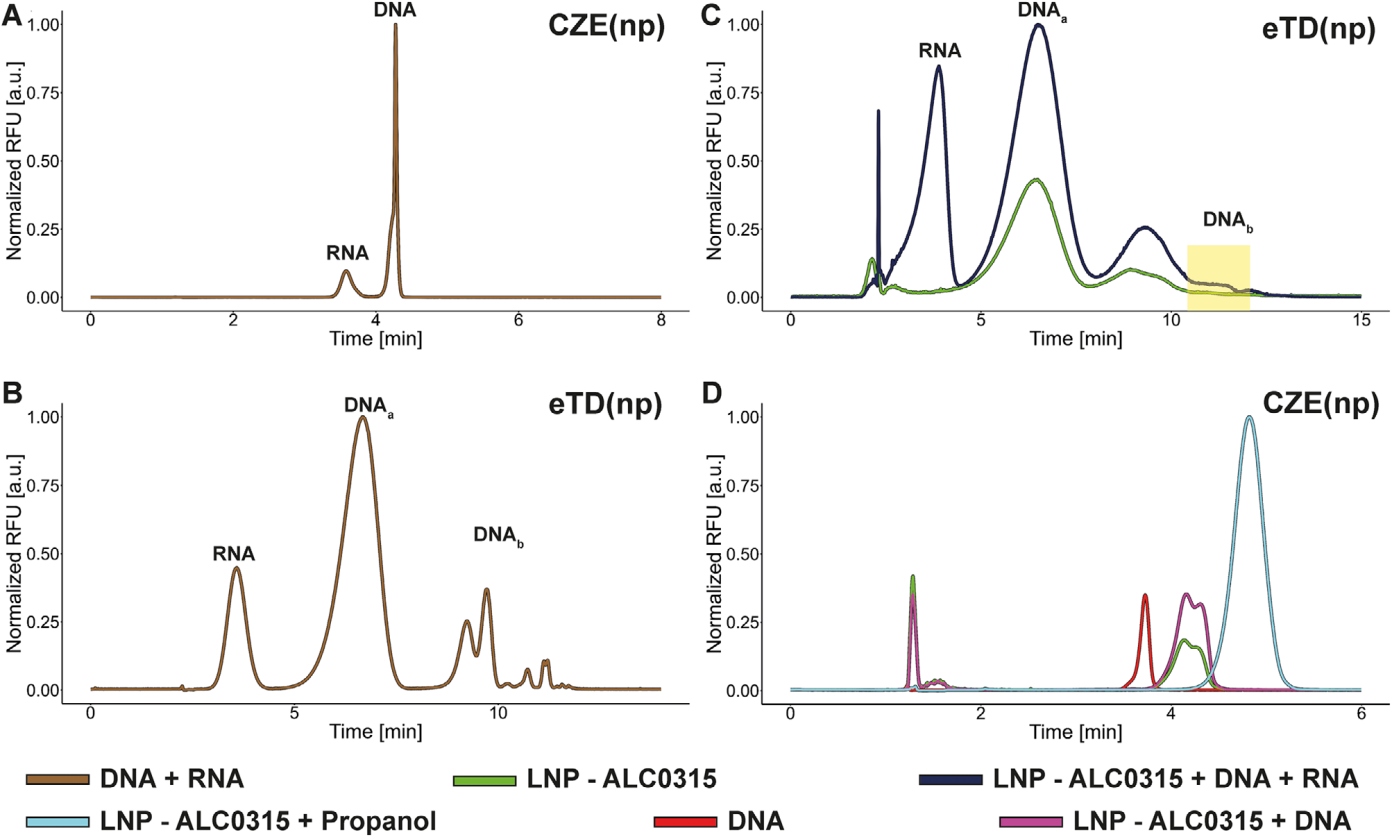
Evaluation of RNA and DNA as an example of ss and ds nucleic acids (free and spiked) and DNA comigration. A mixture of DNA (1 µg/mL) and RNA (7.6 µg/mL) (brown) was analysed using (A) CZE(np) and (B) eTD(np). (C) DNA‐LNPs (green) were spiked with DNA (1.6 µg/mL) and RNA (7.6 µg/mL) (dark blue) and analysed (to note, the yellow box bookmarks the absence of DNA_b_ variants). (D) CZE(np) of intact (green) and spiked (purple, +0.95 µg/mL) DNA‐LNPs (ALC‐0315), of free DNA (red, 0.7 µg/mL), and DNA‐LNP treated with propanol to induce LNP disassembly (light blue). CZE, capillary zone electrophoresis; DNA, deoxyribonucleic acid; eTD, electrophoretic Taylor dispersion or electrohydrodynamic coupling; LNP, lipid nanoparticle; RFU, relative fluorescence units; RNA, ribonucleic acid.

In the initial phase, we investigate the analysis of free DNA and DNA encapsulated within LNPs (Figure 2), illustrating the behaviour of both analytes across four different separation techniques. All experiments employ post‐labelling with SybrGreen. Subsequent studies provide insights into the separation characteristics, the behaviour of various LNP systems, the detection of unencapsulated DNA and changes in separation profiles associated with sample ageing (Figure 3). Finally, we examine the physicochemical properties of single‐stranded RNA (ssRNA) and double‐stranded DNA (dsDNA), both individually and in combination with LNPs, to assess their behaviour under the applied analytical conditions (Figure 4).

**CZE(np)**: As previously mentioned, CZE provides insights into ionic mobility, which indirectly corresponds to the ζ‐potential of the LNPs. Measurements of ζ‐potential using ELS have demonstrated some degree of relevance for the LNP systems under investigation. The absolute values obtained for the individual LNPs by ELS followed the trend: ζ_ALC‐0315_ > ζ_SM102_, ζ_MC3_ by Figure S6. However, the precision of these measurements is limited, and their accuracy is challenging to verify. The ζ‐potential is a key parameter reflecting the electrokinetic surface charge of LNPs, which directly affects their colloidal stability, interactions with biological membranes and transfection efficiency of LNPs [26, 27].

Using CZE under np, clear differences emerge between the profiles of free DNA and the DNA‐LNP formulation (Figure 2A). Both samples were prepared identically, involving the addition of SybrGreen followed by a short incubation, CZE separation and detection by LIF. Both free DNA and the LNP formulation demonstrate anionic migration, with an estimated EOF under the given conditions of approximately 5.2 × 10^−8^ m^2^/*V*·*s*. A notable broadening of the main signal for the LNP system shows that the DNA present in the LNP is in a distinct chemical environment/phase and hence has different migration behaviour compared to free DNA, spanning from near‐neutral charge regions to anionic charges. Additionally, minor signals were detected close to the EOF position, further supporting the complexity of the system. Considering the ionic migration of only the main signal, it can be stated that *µ*_e_ resides between −3.7 × 10^−8^ and −4.1 × 10^−8^ m^2^/*V*·*s*. This corresponds to a ζ‐potential ranging from −47.43 to −52.15 mV. It should be noted that the observed electrophoretic mobility of LNPs in the here presented experiments is comparable to previous work [2]. There, ionic mobility in the range of −3.5 × 10^−8^ to −3.4 × 10^−8^ m^2^/*V*·*s* was obtained for RNA of different lengths.

**TD**: When the sample is eluted through the capillary under applied pressure in TD, the DNA signal manifests as a cone‐shaped distribution with a sharp, pronounced peak at its centre, whereas the LNP signal exhibits a broad distribution accompanied by a negative peak at its maximum. The DNA signal suggests the presence of multiple species with varying hydrodynamic radii. In contrast, the broad LNP signal indicates a predominance of larger size variants, with a negative signal potentially may reflect the fact that the separation takes place in the regime between TD and HDC (Figure 2B).

**eTD(np)**: The combination of an np electric field and pressure‐driven flow results in altered migration patterns for both free DNA and the LNP formulation. In the case of free DNA, a major signal is accompanied by two smaller populations. These minor peaks, exhibiting narrower widths and higher negative mobility, likely correspond to more compact DNA conformers. Importantly, these forms seem to be reversible, with the equilibrium distribution influenced by the chemical environment, such as ionic strength or presence of surfactants (Figure S3). The earlier migrating species—characterized by broader peaks—are presumed to exhibit lower diffusion coefficients, potentially reflecting truncated cone‐like structures observed in TD analyses (Figure 2C). Although CZE experiments show comparable *q*/*r*_h_ ratios for the DNA variants, eTD reveals distinct separation patterns due to hydrodynamic interactions affecting their distribution within the laminar flow. As previously described, under specific conditions, macromolecules can undergo transverse migration here [22], accumulating near the capillary wall or centre and exhibiting correspondingly reduced or enhanced flow velocities.

For LNP formulations the signals are generally broader, with a higher signal detected at the bulk flow time, driven by the combined effects of EOF and applied pressure. Like the findings for the free dissolved DNA, two additional variants appear in the ‘more anionic’ range. Although this suggests a potential correlation, the precise nature of the origin of the individual variants and the relationship to the DNA characteristics remains speculative. Compared to the CZE profiles, it is noticeable that the LNP's effective velocity is accelerated compared to the DNA, probably due to the central focusing within the capillary cross section, which would lead to an increased overall movement.

The influence of the *V*/*p* ratio on the separation profile clearly indicates it to be a decisive factor and confirms the interaction between the characteristics of the parabolic profile and the contribution of the EOF (Figure S2). These investigations revealed that at a ratio of approx. 15–25 kV/psi, the separation profile exhibits several variants, that is, the differentiation of the variants is most pronounced. As soon as the electrophoretic process or the dispersion becomes too dominant, the profile loses its separation.

**eTD(rp)**: When reverse polarity is applied with suppression of the EOF, a different behaviour is observed. Under these conditions, both the bulk flow and analyte migration are directed toward the anode and with EOF suppressed, the parabolic flow profile becomes more pronounced (Figure 2D). The DNA migrates earlier, followed by the broader signal related to the nanoparticles, which means an inversion of migration behaviour. This observation is unexpected, because in the np mode, an acceleration of the nanoparticles was measured, leading to a comigration of the free DNA species and the LNP variants. These distinct migration patterns, compared to np mode, suggest that the DNA is located near the maximum velocity within the laminar pressure‐driven flow (see also spiking experiments shown in Figure S5 and Figure 3C). As already mentioned, a change in direction of the electric field and applied pressure can lead to an adjustment in the migration/dispersion behaviour of the different analytes, depending on their specific hydrodynamic properties. Probably due to the shorter separation time under these conditions, the DNA variants observed in eTD(np) are no longer visible, even though a finely structured signal is preserved.

### 3.4 Evaluation of DNA‐LNP Formulations Considering Individual LNP Systems, Total DNA and Stability‐Indicating Features

In the following study, TD and eTD(rp) are employed to characterize various LNP systems that differ solely in their structural composition, specifically the type of ionic lipids used. TD of DNA‐stained analyte offers a valuable complement to the DLS approach by avoiding the overestimation of larger particles and offering information about the localization of DNA within the formulation. Even if this study does not yet show any final results with regard to hydrodynamic radius, the visual assessment should already provide an initial insight.

From the analysis of the three LNP systems applying DNA‐stained TD (Figure 3A), several conclusions can be drawn. Although the identical peak widths suggest a similar hydrodynamic radius across all systems, confirming the DLS results (Figure S6), the profiles differ significantly in signal intensity and the presence of a negative signal. The total signal areas correlate approximately with the eTD overall peak areas (Figure 3B), indicating that either the DNA concentration within the particles varies or differences in morphological arrangement lead to shielding or accessibility effects that may influence the signal response. We suspect the latter, as the concentration of the total DNA, released by the treatment with propanol, and analysed by CZE is nearly equal for all three LNP systems (Figure S4).

The eTD(np) profiles of the individual LNP formulations are comparable, albeit with individual characteristics, spanning from the neutral to the anionic range (Figure 3B). For the MC3‐LNP formulation, the obtained profile shows more pronounced variants in the later migration range at the expense of the first variant. As mentioned above, the reason for the now altered mobility of the moving particle variants, compared to CZE, in the presence of a parabolic flow remains hidden from us, and even cryo‐EM analysis was unable to provide further clarification. An arrangement of the particle species at different positions in the parabolic flow profile is again the most likely answer where one part accumulates in the maximum flow profile and another part moves closer to the capillary surface. Nevertheless, it reflects the combined electrophoretic and hydrodynamic properties of all DNA‐containing particles present in the test sample solution. Differences in total peak area comparing the three individual LNP systems have already been observed in TD and CZE (see Figure S9), which presumably correlate with different morphological properties of the individual systems. On the other hand, measurements evaluating repeatability (Figure S8) demonstrated reproducible signals in terms of both profile and intensity.

The application of eTD(rp) offers the potential to assess the presence of free, unbound DNA (Figure 3C). Unlike eTD(np), the LNP species migrating between 4 and 7.5 min are not well‐separated in this mode. The signal group with the latest elution/migration time corresponds to the neutral point and bulk flow. Notably, free DNA is detected first in the migration order, distinctly separated from other signal groups. This observation was validated through spiking experiments with free, unbound DNA (Figure S5) and further confirmed by analysing an LNP sample degraded with propanol (Figure 3C). Consequently, the signal observed between 2 and 3 min of the intact LNP solution can be attributed to ‘free DNA’ or DNA‐lipid complexes that are not part of the LNP particle.

But how reproducible and comparable are the separation profiles when aged LNP formulations are analysed, and could the eTD(np) be used for stability‐indicating features? A freshly prepared test sample was analysed and compared to the same analyte stored for 1 month at 8°C. Indeed, a growth of the later migration variants was observed across all LNP systems, highlighting the potential of eTD(np) for detecting stability‐related changes (Figure 3D). This alteration appears to occur gradually, reaching a steady state after approximately 2 months when the LNP systems are stored at 8°C. Cryo‐TEM analysis of the aged LNP formulation did not show any abnormalities or distinct changes compared to freshly prepared samples that could be associated with the changed eTD profile. However, it should be noted that with cryo‐TEM only a small evaluation window can be observed and never presents an overall picture. We suspect that these changes are associated with a realignment or alterations in particle structure and morphology, which may also affect their size. For all three LNP types, an increase in the hydrodynamic radius analysed with DLS was observed (Figure S7).

### 3.5 Migration Behaviour and Identity of ss and ds NA

In this section, we first discuss the migration behaviour of free, dissolved ss and ds NAs. This is particularly relevant when distinguishing between the two forms is essential, such as in the identification and relative quantification of dsRNA contaminants in mRNA vaccines. Current preferred methods for RNA transcript characterization include UV spectroscopy, fluorescence‐based assays and immunoassays [28]. However, these techniques have notable limitations, including low resolution, complex handling, the use of hazardous reagents, labour‐intensive protocols, reliance on antibodies, lengthy run times and scalability challenges [29, 30]. An innovative analytical approach utilizing gel CE, referred to as CGE conducted in capillaries, was introduced by Coll de Peña et al. This method offers a useful option to indicate dsRNA and ssRNA [31]. In contrast, CZE used in this study does not require gel‐containing BGE, allowing for the determination of total NA concentration without the risk of co‐migration of ssRNA and dsRNA, even in cases of fragmentation.

As previously noted, CZE cannot effectively resolve NAs that differ solely in chain length, as their charge‐to‐hydrodynamic radius (*q*/*r*_h_) ratios are nearly identical at a pH of 8. Nevertheless, CZE offers a simple and efficient method for simultaneously assessing the presence of ss and ds NAs. Due to differences in secondary structure and consequently in hydrodynamic radius and effective charge, dsNAs are expected to exhibit slightly higher negative electrophoretic mobility than their ss counterparts. This migration behaviour was confirmed through simultaneous analysis: RNA migrated earlier than dsDNA, consistent with its lower anionic mobility (Figure 4A). Although the injected RNA sample had a higher mass concentration, the fluorescence response is reduced as expected based on differences in dye binding. Accurate determination of absolute concentrations and relative proportions can be achieved through calibration with defined dsRNA and ssRNA reference materials.

Figure 4B presents the resulting eTD(np) profile of the same mixture analysed by CZE. As expected, the RNA migrates/elutes earlier, comparable to the CZE(np), and only one signal can be identified. The resulting profile of the DNA has already been discussed, confirming the reproducibility of the separation. Regardless of the length of the NAs to be investigated, the same behaviour is expected when ss and ds NA are analysed together.

When DNA and/or RNA are added to an LNP formulation and analysed using eTD(np), a test sample solution is generated that contains both encapsulated NAs and free, unbound species (Figure 4C). The resulting migration profile closely resembles that of the unspiked LNP formulation, except for an additional RNA‐associated signal and an overall increase in signal intensity. The DNA_a_ signal group, previously observed at lower migration times in the analysis of free DNA (Figure 4B), co‐migrates with the main peak of the LNP formulation. In contrast, the DNA_b_ group, detected at higher migration times in the free DNA sample, disappears in the spiked formulation, appearing to merge with signals associated with LNP variants. This observation may reflect micelle‐like DNA structures external to the nanoparticles. As a result, under the present separation conditions, quantitative assessment of non‐encapsulated DNA is not feasible. In contrast, eTD(np) appears well‐suited for the detection and quantification of unbound, non‐encapsulated RNA, highlighting its potential utility in RNA‐specific LNP formulations. In a last overview (Figure 4D), overlays are summarized and normalized to the largest signal, corresponding to the profiles of intact and DNA spiked LNPs as well as the profiles of free DNA and after treatment with propanol. So far, only the profiles have been discussed; now, the direct comparison regarding the detector response confirms that when the total DNA is released, the signal intensity multiplies due to the lack of shielding.

### 3.6 Critical Quality Attributes

The International Council for Harmonization of Technical Requirements for Pharmaceuticals for Human Use recommends in its guideline ICH Q11 that critical product attributes, such as physical characteristics, should be defined and monitored during manufacturing to ensure consistent product quality and performance. Indeed, physicochemical characteristics depend on critical process parameters during manufacturing, which, in turn, influence biological efficiency. For example, LNP morphology is affected by the mixing ratio and speed of mixing of the ethanol‐lipid phase to the mRNA aqueous phase [32]. It is, therefore, tempting to speculate that the proposed analytical tools could be used for monitoring of LNP‐based products during manufacturing. Analysing sample volumes of a few microlitres, product identity and overall quality could be assessed qualitatively based on their electrohydrodynamic coupling fingerprint. Using the same analytical device and test sample solution, subsequent analysis by CZE, TD and eTD can be applied to assess quantitatively parameters of interest such as hydrodynamic particle size, size distribution, NA loading efficiency and electrophoretic mobility of LNPs that correlates with the ζ‐potential of the particle.

## 4 Concluding Remarks

The characterization of novel LNP formulations requires adaptability and innovative thinking, particularly when utilizing advanced analytical tools. In this study, we present one entirely new separation principle, eTD analysis, alongside two established techniques that are applied here for the first time to the analysis of LNPs stained with a DNA‐specific dye. Although these methods are not yet fully standardized, they provide a strong foundation for the development of robust analytical approaches to complement existing techniques. The goal is to enable the safe, reliable and comprehensive characterization of diverse LNP systems in the context of a CQA determination.

First and foremost, the migration behaviour of various LNP systems in an electric field (CZE) was analysed and compared to that of free, dissolved DNA. This analysis was subsequently extended into a migration–dispersion‐based technique, termed eTD, allowing the maintenance of the chemical equilibrium. Both approaches yield valuable insights: Although the ionic migration behaviour of LNPs and DNA is comparable, LNPs exhibit slightly higher negative ionic mobility. The application of additional pressure results in an accelerated effective migration of LNP, likely attributed to the influence of the parabolic flow profile generated under these conditions. However, this phenomenon complicates the simultaneous analysis of LNPs and free DNA. Notably, although different LNP systems produce comparable eTD(np) profiles, they display variations in absolute signal intensity and the peak area ratios of individual signal groups. These distinctive profile characteristics serve as a foundation for the detailed characterization of LNPs. In particular, the new method allows for a qualitative comparison of LNP preparations to monitor batch‐to‐batch differences in production or to support stability studies.

The analysis of unformulated RNA and DNA yields unambiguous results. First, double‐stranded DNA exhibits a higher ionic mobility compared to single‐stranded RNA, which can be attributed to its double charge per NA unit, as observed in both CZE and eTD. Thus, both CZE(np) and eTD(np) prove to be highly suitable for the determination of ds and ss NAs with excellent sensitivity. Second, eTD(np) enables the detection of DNA conformations (i.e., linear, circular and supercoiled), which could hold relevance for quality control of raw materials or formulation studies. To assess the unencapsulated portion of RNA or DNA present in LNP formulations, it should be noted that different protocols have to be used for the analysis. Our experiments showed that eTD(np) is the preferred method for RNA‐LNP systems, whereas eTD(rp) is more advantageous when DNA is the focus.

TD serves as a valuable complement to the standard DLS technique. TD's signal intensity and peak width provide insights into the concentration of NA and/or accessibility of an NA dye and the size distribution of particles. Notably, TD can also be applied for freely dissolved NAs. As mentioned, the analysis of DNA using TD revealed unexpected patterns, suggesting the presence of multiple conformations, which were further confirmed using eTD(np).

Looking ahead, it can be summarized that the separation methods presented can already be applied for the characterization and quantification of free NAs. Analysing LNP systems for TD still lacks suitable commercial equipment and the ability to analyse the data accordingly using the commercially available CE devices. The systematic investigation of operational parameters such as pressure, capillary diameter, particle size or differentiation between LIF (DNA sensitive) and UV detection, but also the clever implementation of lipophilic dyes, will provide a deeper understanding of the dispersion characteristics of LNPs, visualizing the lipid‐containing phases. With this knowledge and the knowledge of the intrinsic electrophoretic behaviour, electrohydrodynamic coupling can then be better studied and understood. For this purpose, systematic investigation of the parameters mentioned and additional operational parameters, as the electric field, the strength of the EOF and the relationship between pressure and voltage, are of interest.

Furthermore, future research should aim to explore other types of LNPs or alternative NA‐based carrier systems to provide mechanistic insights into the behaviour of the complex nature of considered drug formulation systems. Optimization of the presented separation principles will ultimately allow for a qualitative and quantitative assessment of CQAs of nanoparticles. Finally, it should be noted that electrohydrodynamic coupling may generally be extended to other particles of viral and synthetic origin, presumably also to other larger biomolecules such as conjugated proteins, plasmid DNA or other NA constructs.

## Conflicts of Interest

The authors declare no conflicts of interest.

## Supporting information




**Supporting Information File 1**: elps70032‐sup‐0001‐SuppMat.docx

## Data Availability

The data that support the findings of this study are available from the corresponding author upon request.
