# Supplementary material for: Capillary‐Based Physicochemical Characterization of Lipid Nanoparticles
Source: Electrophoresis. 2025 Sep 28;46(21):1588–99. doi: 10.1002/elps.70032 (PMC12595287; doi:10.1002/elps.70032)

# Supporting information


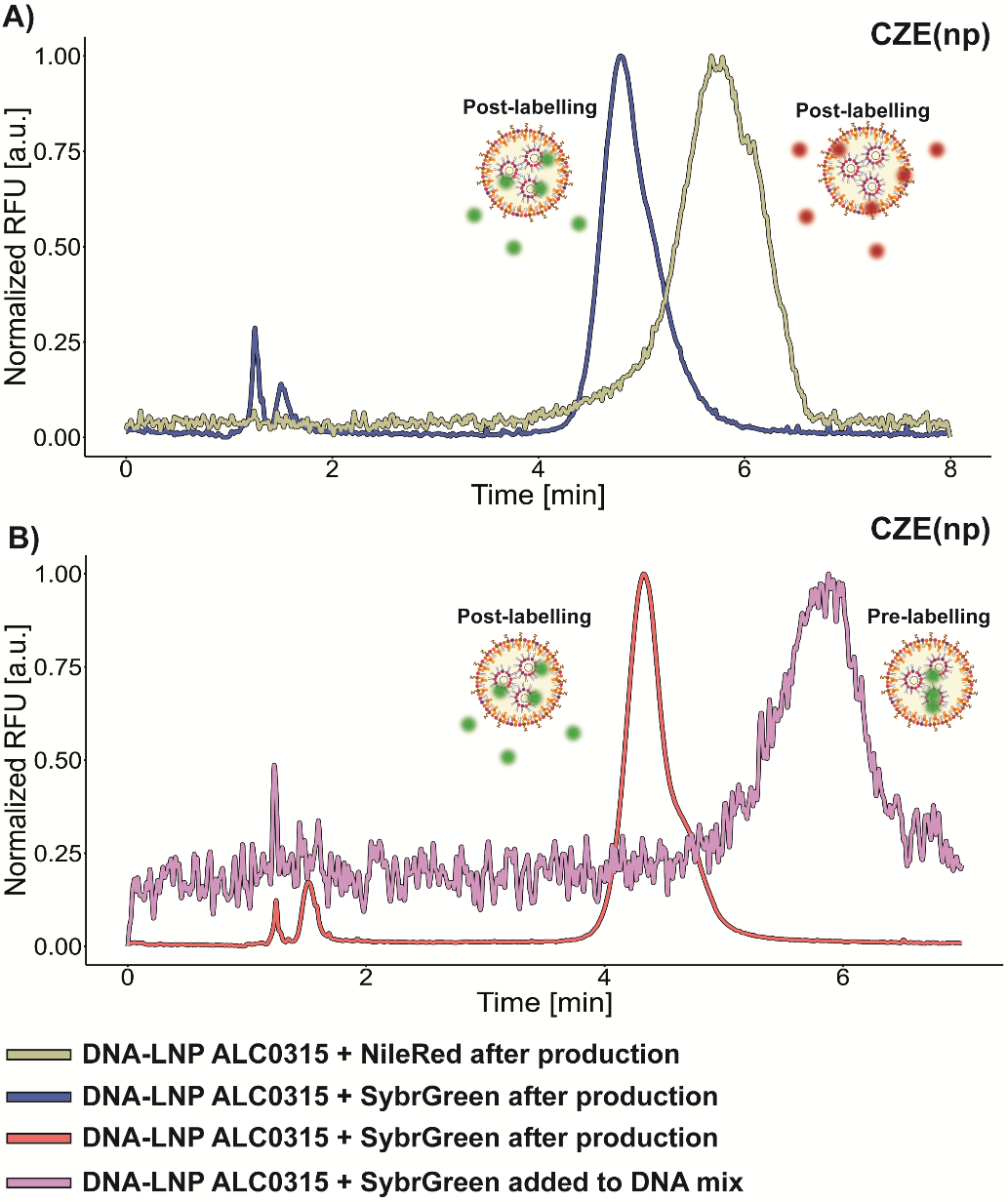
***Figure S1. The identity of lipid and DNA containing phases.*** *(A) Comparison of CZE profiles of DNA-LNPs stained via their lipid components using the lipophilic dye NileRed (NR, green) and according to standard method (blue). (B) Comparison of CZE profiles of DNA-LNPs pre-labelled during manufacturing with SybrGreen (pink) and according to standard method (red).*

Both SybrGreen and NR were incubated during sample preparation and analyzed using CZE(np), with SybrGreen also included in the BGE for the SybrGreen -stained sample. The signal response for NR was significantly lower, while the effective ionic mobilities of the two dyes were comparable (SybrGreen /NR: 4.12/4.35 × 10^-8^ [m²/V·s]). However, staining with SybrGreen resulted in a slight but systematic reduction in ionic mobility. This phenomenon could be attributed to the strongly basic nature of SybrGreen, which may influence ionic migration toward the cationic direction.

Comparing the test sample in which SybrGreen was incorporated during manufacturing, either before formulation or prior to dialysis, with a test sample analyzed according to the standard protocol, the signal intensity significantly reduces but also the migration time is slightly altered. This is likely due to stronger spectral shielding, possibly resulting from SybrGreen being positioned more centrally within the LNP structure at a lower concentration. The shift in migration time can be explained by the absence of SybrGreen in the BGE and test sample solution.


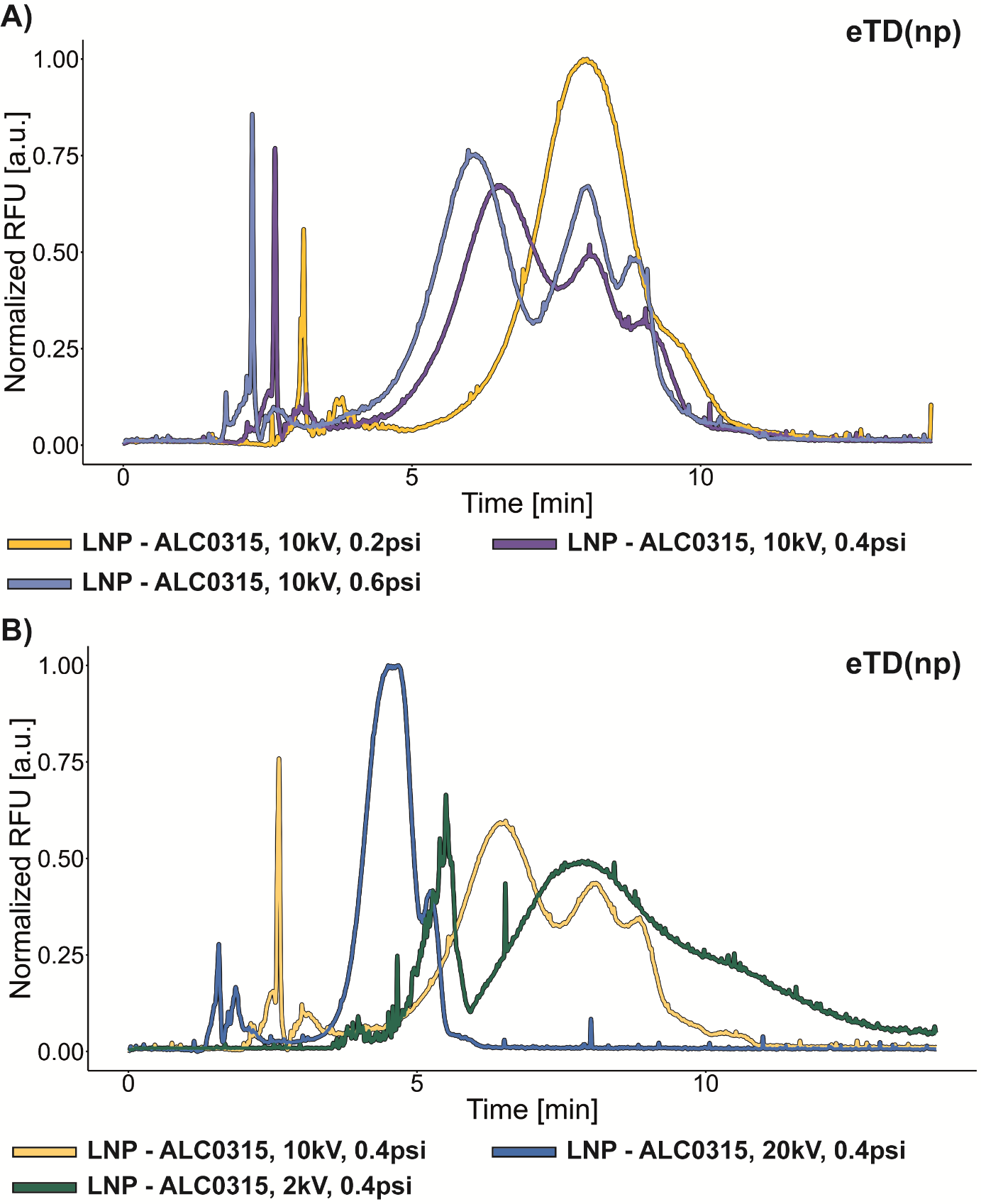


***Figure S2.*** ***Influence of electrokinetic transport parameters on the migration behaviour of DNA-loaded LNPs analyzed by eTD(np).*** *Electrophoretic traces illustrate the effect of varying pressure and voltage conditions. In the upper panel (A), a constant voltage of 10 kV was applied while the pressure was incrementally varied between 0.2 and 0.6 psi. In the lower panel (B), the pressure was fixed at 0.4 psi, and the applied voltage was varied from 2 to 20 kV. Other experimental parameters were held constant across both conditions. Distinct migration profiles emerged under different conditions.*


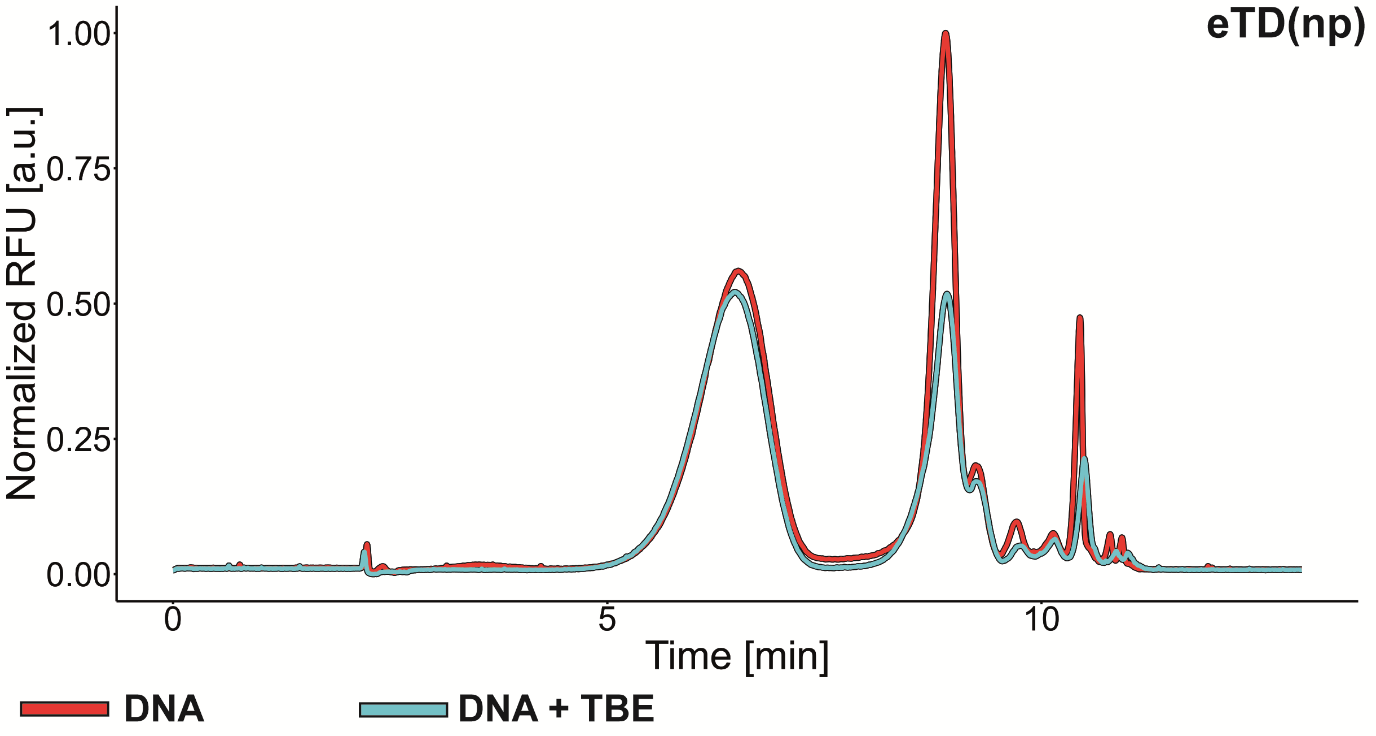
***Figure S3: eTD(np) profiles of free DNA with different diluents used for test sample preparation.*** *DNA diluted with water (red), and 1xTBE (blue), final concentration 0.5 μg/mL.*


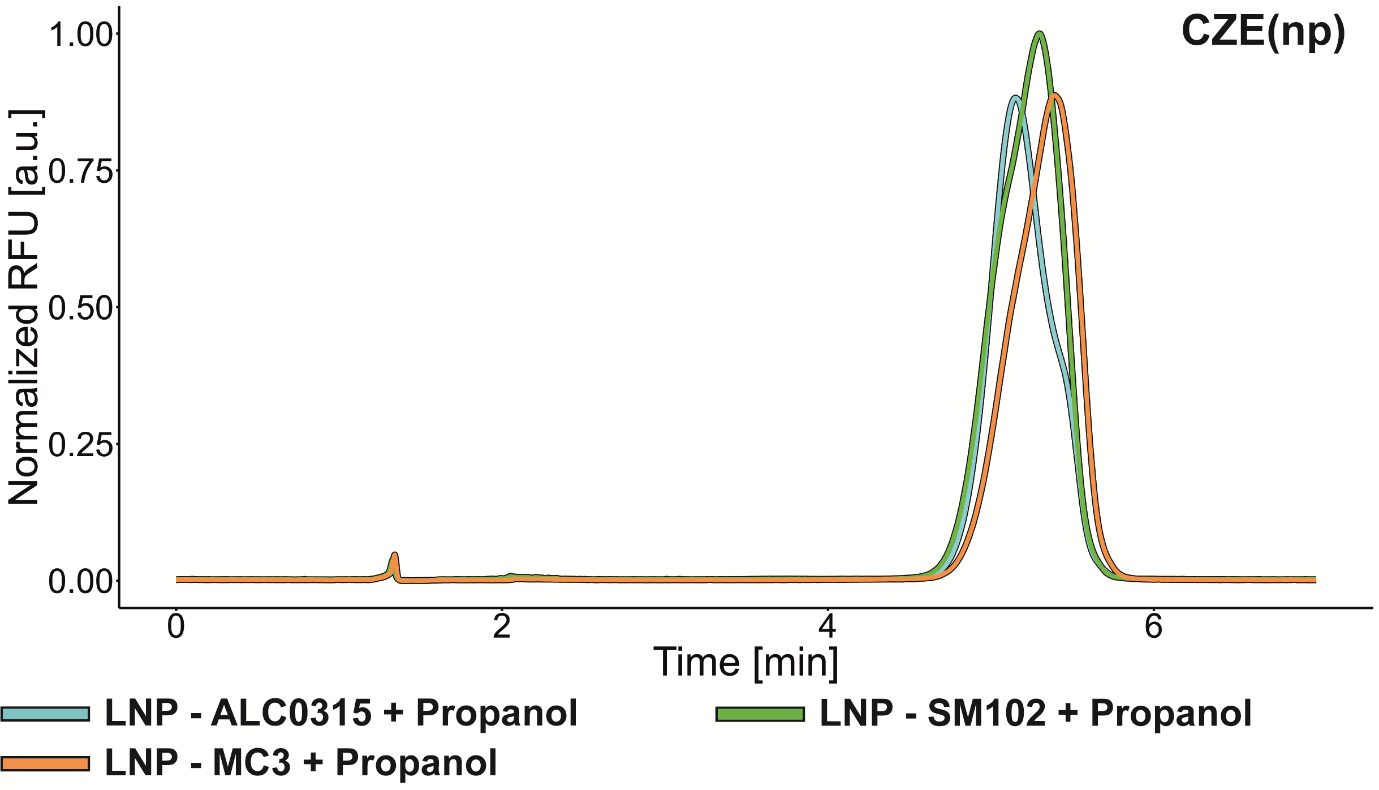
***Figure S4.*** ***Propanol-induced disassembly of DNA-loaded LNPs****. CZE(np) traces of propanol-treated DNA-LNPs show a single, well-defined peak corresponding to released, total DNA. The peak intensity is consistent across tested formulations, indicating complete disassembly of the LNPs.*


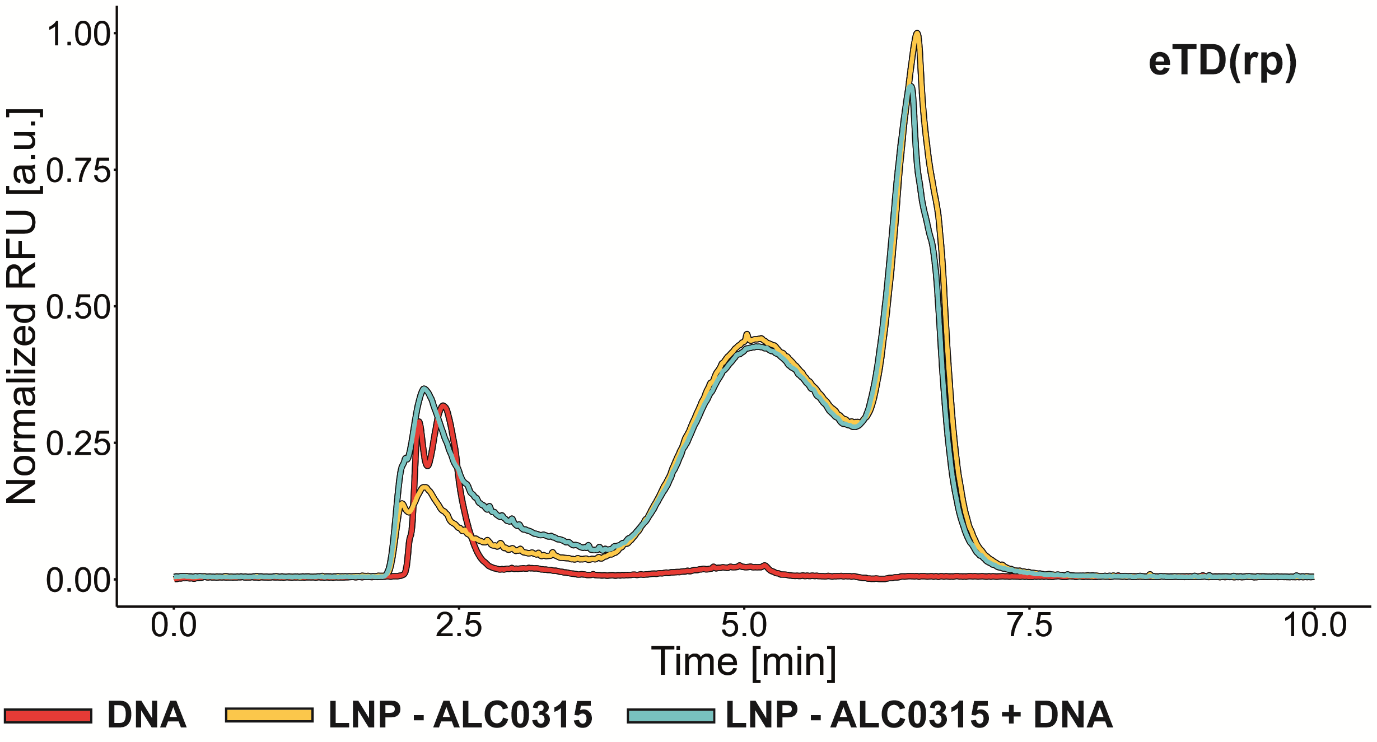
***Figure S5.*** ***Detection of free DNA in DNA-LNP samples using eTD(rp)****. Electrophoretic traces compare free DNA (red, 0.8 μg/mL), DNA-LNPs (yellow), and DNA-LNPs spiked with free DNA (cyan, + 0.8 μg/mL). A distinct migration feature appears at approximately 2.5 minutes in samples containing free DNA, enabling clear differentiation between encapsulated and unencapsulated DNA within the system.*


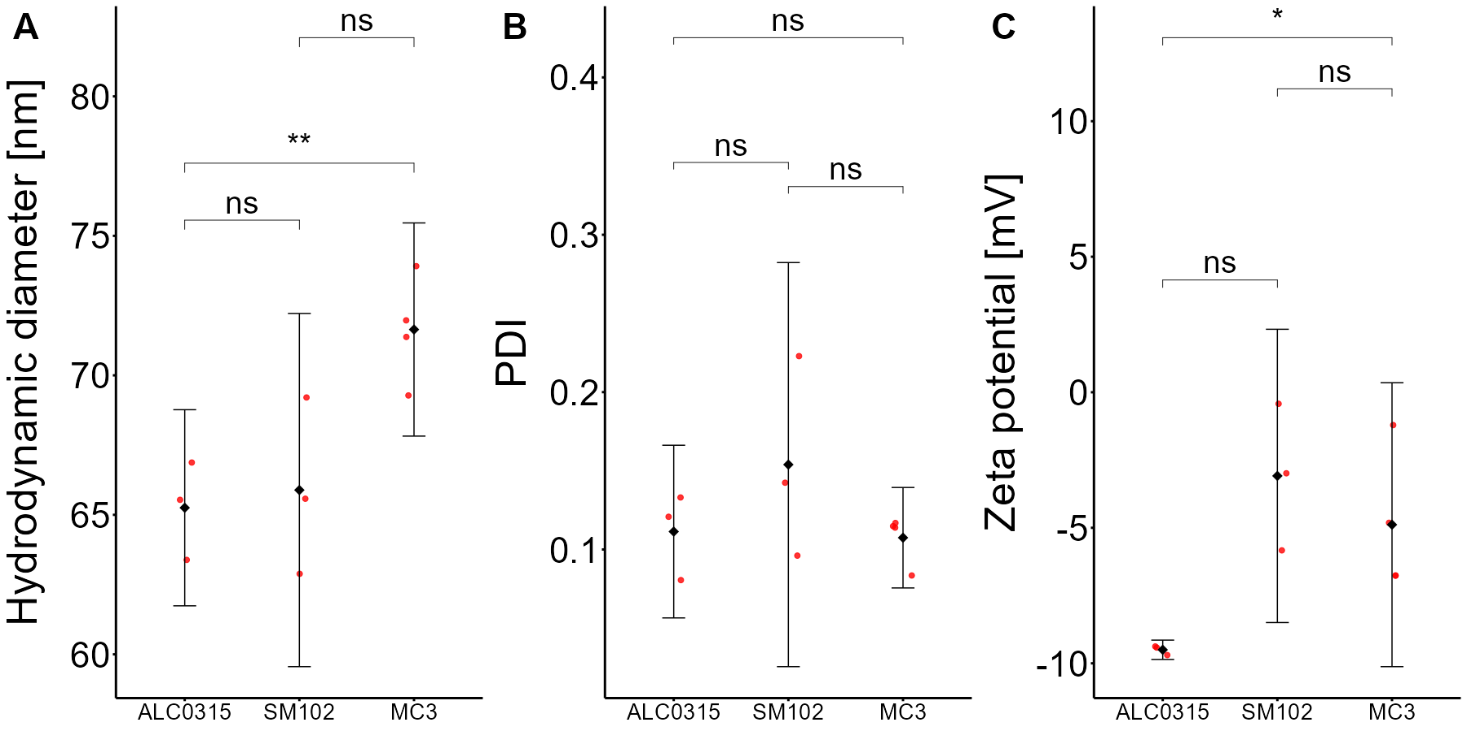


***Figure S6.*** ***Physicochemical characterization of DNA-loaded LNPs by DLS and ELS.*** *(A) Hydrodynamic diameter, (B) PDI, and (C)* ζ *is shown for each DNA-LNP formulation. Black dots indicate the mean values from biological replicates (n = 3 for ALC0315 and SM102; n = 4 for MC3). Red dots represent individual data points; whiskers denote standard deviation. Statistical significance was assessed using an unpaired Welch’s two-sample t-test with unequal variances. p-value thresholds represent ns (p > 0.1), * (0.01 < p ≤ 0.05), ** (0.001 < p ≤ 0.01).*


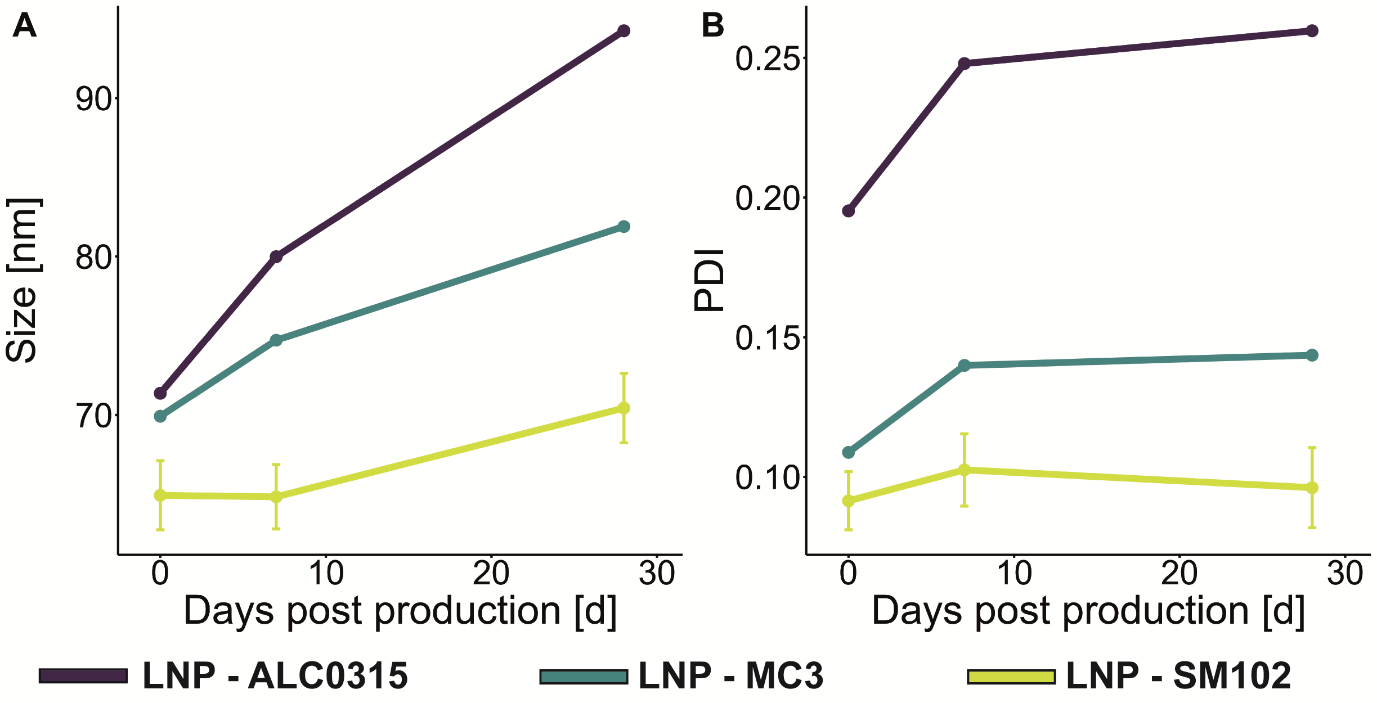
***Figure S7****.* ***Size (A) and PDI (B) results of aged test samples.*** *LNPs were evaluated at the day of production (t = 0 days), after a week (t = 7 days) and after a month (t =28 days). For LNP-ALC0315 and LNP-MC3 one formulation batch (n = 1) was evaluated (no error bars shown), for LNP-SM102 three formulation batches (n = 3) were evaluated. Error bars represent the standard deviation per time point.*


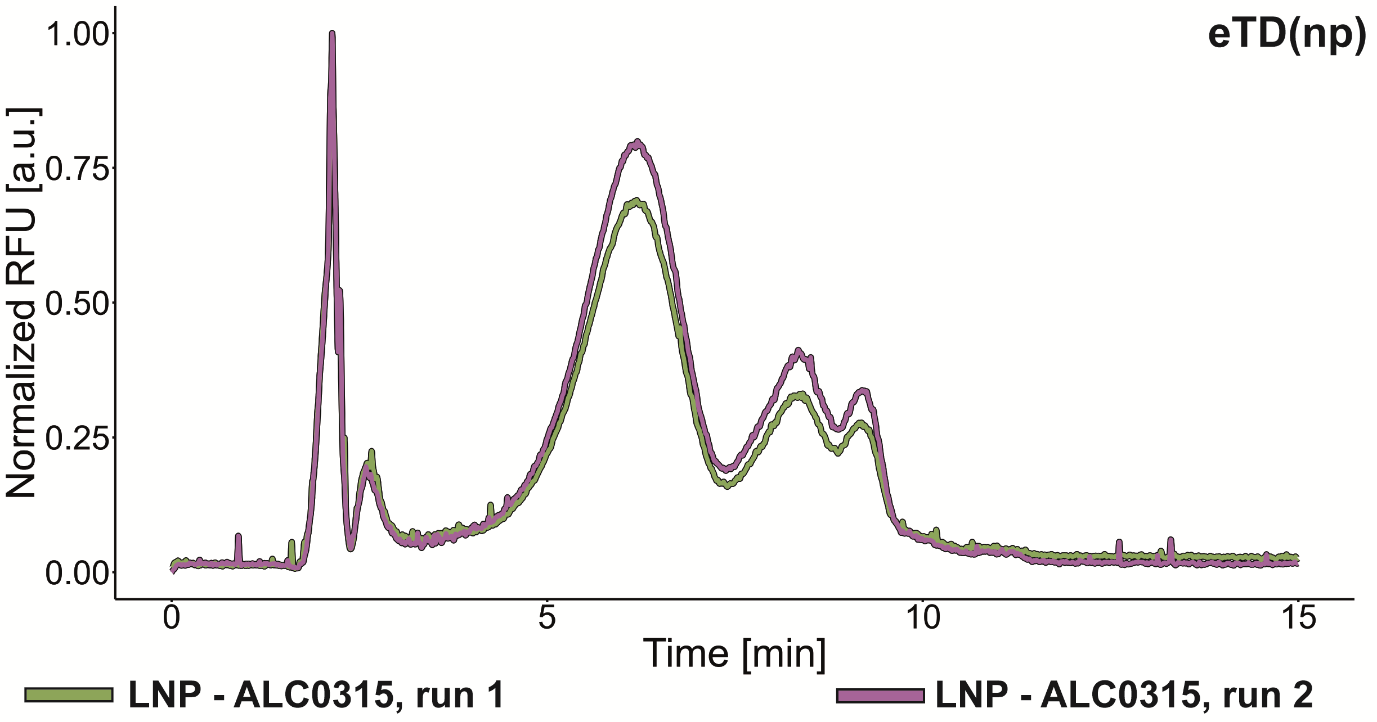


***Figure S8.*** *Reproducibility of two different test sample preparations of the same LNP-ALC0315 formulation. eTD(np) standard conditions were applied.*

**Figure S9**. CZE(np) was applied on different LNP-formulations with various ionizable lipids. Same experimental conditions as in Figure 2A.


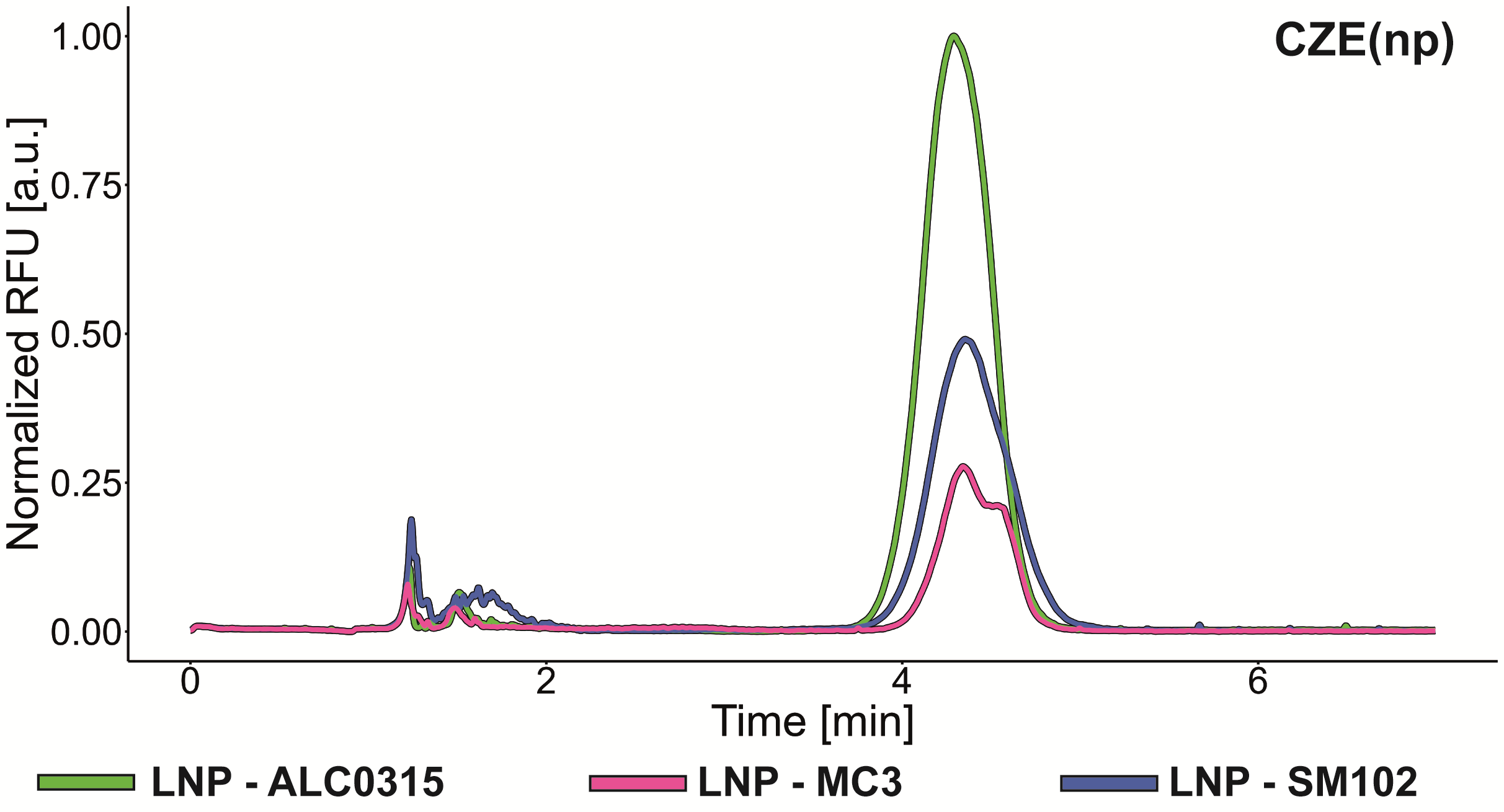

Supplement: Supplementary file 1 — Supporting Information File 1: elps70032‐sup‐0001‐SuppMat.docx [file ELPS-46--s001.docx]
